# The need for a comprehensive diabetes data system in Ireland

**DOI:** 10.1111/jdi.70080

**Published:** 2025-05-27

**Authors:** Naomi Holman, Claire M. Buckley, Lisa Devine, Kate Gajewska, Sean F. Dinneen, the Chronic Disease Data System Consortium, Edward Gregg, Declan Noone, CONVERGE Research Group, Lauren Churchill, Karina Hamilton, Collette Tully, Austin G. Stack, Leonard Browne, Patricia Kearney, Sandhya Rengarajan, RECONNECT Research Group, Jennifer Martin, Fionnuala Donohue, Majella Daly

**Affiliations:** ^1^ School of Population Health Royal College of Surgeons in Ireland Dublin Ireland; ^2^ School of Public Health Imperial College London UK; ^3^ School of Public Health University College Cork Cork Ireland; ^4^ National Health Service Improvement Team Health Service Executive Dublin Ireland; ^5^ Irish College of General Practitioners Dublin Ireland; ^6^ Division of Advocacy and Research Diabetes Ireland Santry Dublin Ireland; ^7^ Discipline of Medicine University of Galway Galway Ireland; ^8^ Centre for Diabetes, Endocrinology and Metabolism Galway University Hospitals Galway Ireland

## WHY IS A DIABETES DATA SYSTEM NEEDED IN IRELAND?

The primary purpose of healthcare systems is to prevent and treat disease. The mainstay of this is direct patient clinical care, but the system needs to be supported by reliable and timely data to function in an evidence‐based and responsive way^1^. Many health systems around the globe have a rich tradition of using data to inform and improve care processes^2^, ^3^, ^4^. In Ireland, there is currently no single source of data that can reliably report the number of people living with diabetes and no collation of records that provides an overview of the care received and outcomes experienced by people living with diabetes. A dual payer (public/private) system has led to diverse models of care, with a substantial proportion of diabetes care being provided privately by general practitioners. Historically, the lack of a unique identifier makes linkage of (clinical and administrative) data systems challenging. Many questions remain unanswered, and the scope to improve services and health‐services planning is hindered without mechanisms to monitor change. Issues of equity and equality remain hidden, and the perspectives of people within the health service on the characteristics and outcomes of people living with diabetes cannot be substantiated by data.

Without a comprehensive data system, there is no scope to verify (or disprove) perceptions and changes to services that may be made with limited data to support their evaluation; plans are not evidence‐based, and data to prioritize and shape policymaking are lacking.

## WHAT IS THE CURRENT POSITION OF DIABETES RELATED DATA IN IRELAND?

**Figure 1:**
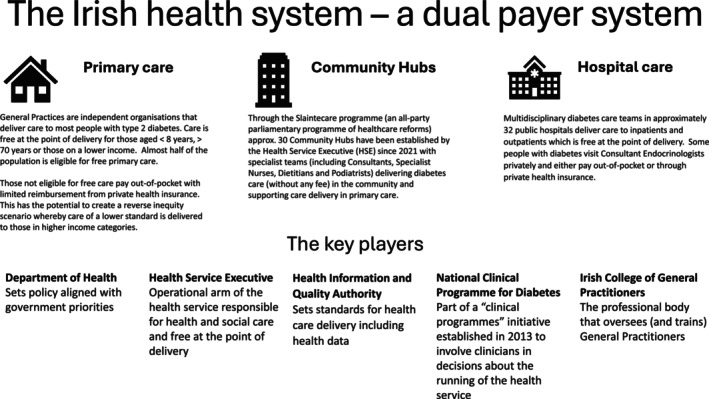
Schematic overview of the Irish health system.

Currently, aspects of diabetes care are captured in several national administrative datasets, including records of prescriptions dispensed by community pharmacies, publicly funded hospital admissions, Chronic Disease Management (CDM) programme returns by General Practitioners, Diabetic RetinaScreen, and civil death registrations. The combination of public and private care provision in the Irish health service (Figure 1) means that coverage of the datasets is not always universal. Data analysis is limited by a lack of governance, infrastructure, and public engagement that facilitates continuous networked dataset linkage. A national individual health identifier (IHI) has been created, but full implementation requires further legislation. Additionally, some crucial data on the care and outcomes of people with diabetes, such as laboratory tests, anthropometric measures, and lifestyle risk factors, are held in local or sub‐national systems, which vary in their compatibility. The need for a diabetes data system has been consistently identified and has the support of the Department of Health, the Health Service Executive (HSE), Diabetes Ireland (national patient organization) and academic organizations. With early funding allocated and posts being filled in 2025, processes are being established to provide clinical leadership, programme management, data management, analysis, and reporting functions.

## WHAT IS THE DIABETES DATA SYSTEM AND HOW WILL THE DATA SYSTEM BE USED?

**Figure 2:**
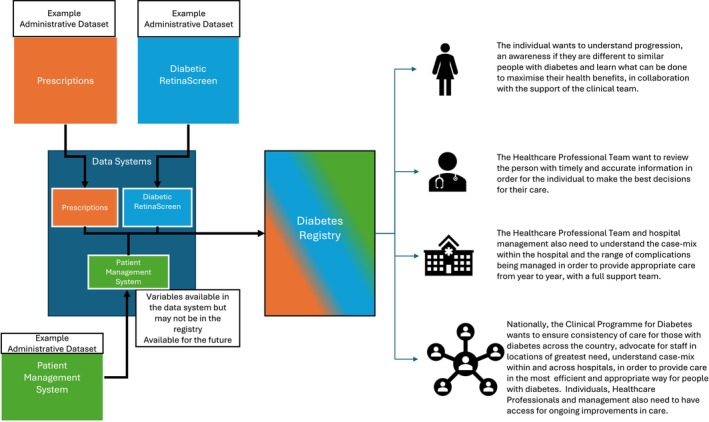
Synopsised example of short and potential long‐term merging of datasets into a data system, to allow appropriate (multi‐level) analysis of the care being delivered to people living with diabetes.

To address the gap in data on diabetes care in Ireland, the HSE initiated a programme of work to create a diabetes data system. It will collate administrative data currently used within the health system to identify people with diagnosed diabetes and document their healthcare delivery and outcomes. It aims to be an exemplar that can be applied to other chronic diseases (Figure 2).

By utilizing existing datasets, the diabetes data system will minimize the burden for clinical staff. It will include the scope to assess the incidence and prevalence of diabetes, the level and variation in care received by people with diabetes, leading to an understanding of equity and equality in healthcare. It will become dynamic by supporting service activity metrics, quality assessment/awareness, improvement of services to enable planning, monitoring, and evaluation, and the generation of new knowledge through scientific methodologies. As the role of real‐world data in academic research grows, the data system will provide a rich dataset to explore the epidemiology and health economics of diabetes. In the long term, there is an aspiration of becoming a learning health system moving toward a real‐time clinical and administrative data system accessible at the point of clinical care.

## WHAT IS THE WAY FORWARD?

The journey from concept to a fully functioning data system will take many steps. A governance structure is needed to implement and oversee the work programme. The formal structure should include a senior leadership group that sets, guides, and monitors the strategic direction of the health service. An implementation group is required that will focus on the delivery of the data system; appropriate analysis and engagement with the diabetes community is also needed. At all levels, the governance system should be able to make decisions, solve problems, maintain support for the development of the data system, and ensure it meets its specified functions. As described in Figure 2, the function of the data system will vary across user groups. The clinical community requires data to assess and understand the level of care provided, allowing initiatives to maximize the quality of care. Service planners need to understand current service provision, how this varies across patient groups, and how future needs may change. People living with diabetes need access to data that allows them to make informed decisions and maximize their care.

The definition and identification of people with diagnosed diabetes needs to be specified. This will be an iterative process where individuals are identified from existing datasets including the Diabetic RetinaScreen programme, the CDM programme, records of prescriptions dispensed in the community through the primary care reimbursement service (PCRS), and hospital admissions (where diabetes has been identified) through the hospital inpatient enquiry (HIPE) database. As the system matures and incorporates data from local sources such as general practice and laboratory records, this process will evolve.

Patient and public involvement needs to be embedded in the programme from the earliest stages. There needs to be a clearly defined process for ensuring that people living with diabetes have a real voice in how the data system develops and that any data analysis has a positive and timely impact on their quality of life. This must acknowledge that there is no single perspective, and capturing the range of views and experiences is vital to ensure the support of the people who form the dataset and for whom the data system seeks to create improvement.

Although the primary constituent datasets are already known, a data discovery and mapping exercise will be required, and the information governance framework to facilitate the shared storage, linkage, analysis, and reporting will need to be created. This will require clearly defining the function of the data system, the scope of analyses to be undertaken, and ensuring appropriate disclosure control measures are in place. Processes to allow the sharing of data need to be incorporated into the framework to ensure the data system can perform its intended functions. To maintain data integrity and provide greatest flexibility to meet currently identified and unanticipated needs, the component datasets contributing to the data system should be stored as regularly updated standalone datasets. Information from each dataset will be pooled to identify individuals with diagnosed diabetes, and an index file will contain their key demographic characteristics, diagnosis information (e.g. type of diabetes) and identifiers to link to specific datasets in the system. A standard suite of data quality checks and data cleaning processes should be developed for each dataset included in the data system^5^.

The foundations of the data system are routinely collated national administrative datasets, so there needs to be scope for it to develop over time and with the flexibility to evolve both in terms of inputs (datasets) and outputs (reports, indicators, bespoke analyses). Regional and local information, population, and disease‐specific information need to be incorporated in the future.

There needs to be a scoping exercise to create a consensus on the nature of outputs from the data system. The fundamental principles are that all outputs are tailored to their audience, data are presented with appropriate context, caveats, and interpretation. Policymakers will require summary‐level reports that provide evidence on the current epidemiology of diabetes, the care received by people with diabetes, how treatments and outcomes vary, and provide insight into future requirements of the diabetes community. National reports with complementary local summary data/indicators focusing on clearly defined measurable factors will facilitate quality assessment and improvement. There will be a need to create easily accessible reports for lay audiences where findings are distilled into clear messages in non‐technical language presented in the wider context with further support signposted. If the data system is to contribute to the generation of new knowledge, it must include a secure way for analysts and researchers to access individual‐level data to undertake appropriate statistical analysis and for dissemination through academic routes such as conferences and peer‐reviewed journals.

Clear, knowledgeable and decisive leadership from clinical and data perspectives will be vital for the development and success of the data system. Integration of expertise from these areas, from the beginning should ensure that it can meet the needs of the diabetes community (including healthcare professionals working in diabetes) and encourage confidence in the data processing, analysis and presentation. Part of this role will be to manage expectations of the diabetes clinical community and among people with diabetes of the scope and capability of the data system. Resources of this nature take time to evolve into comprehensive datasets that can provide a comprehensive picture of care and outcomes. They also need to have dedicated long‐term resources and staff allocated and firmly embedded within the core health service rather than perceived to be an ‘add‐on’ to clinical care. The Diabetes Data System aims to be one of the exemplars for other chronic diseases in Ireland.

Outputs from the diabetes data system may highlight variation in care and outcomes which will require careful interpretation. The process of assessing, questioning and action planning should not be under‐estimated. Ireland does not have a tradition of providing dashboards to diabetes clinicians or people living with diabetes as a way of motivating them to focus on certain aspects of their practice (or behavior). It is therefore vital that there is support in the interpretation of the outputs from the data system for clinical and patient communities. This must include insight into how the demographic and socio‐economic characteristics of individuals with diabetes are (or are not) associated with outcomes. As the foundation of the data system is nationally collated administrative datasets the production of reports and summary measures for and relevant to local health systems creates a circle of feedback to the origins of the data. The accuracy and quality of the data being input need to be reflected in the outputs from the data system. There also needs to be an understanding that timely real‐world data of the sort to be created in the data system rarely provides neat and clear cut ‘answers’ to simple questions. In fact, analysis often leads to more questions being asked as people consider the nuances behind and implications of summary figures. Guidance on unpicking the implications of the data and how to navigate service improvement will be essential to the acceptance and success of the data system.

As the consortium leading this work has evolved organically, it benefits from strong support from senior leaders in clinical, academic, and patient organizations. However, the scale and scope of the task are sizeable. The Irish health system needs a standardized approach to the generation of data systems for diabetes and other chronic conditions. In today's world, data is more readily available, and it is incumbent on us to use data to improve population health. In September 2024, at the time of the Annual Meeting of the European Association for the Study of Diabetes, the European Diabetes Forum (a pan European non‐profit multi‐stakeholder organization advocating for the translation of research into policy) held a workshop on diabetes registries. Many of the (12–15) national or sub‐national registries represented at the workshop had years (or decades) of experience in leveraging change in policy and practice through outputs from robust chronic disease data systems. Ireland was represented at the workshop, and the ambition (described in this article) to establish a diabetes data system for collating, analyzing, and using existing administrative and clinical data to improve the quality of diabetes care for children and adults was acknowledged. Just like the St Vincent Declaration in the 1990s^6^, this call to action to streamline and harmonize diabetes data systems across Europe should be a motivator to the Health Service Executive and partner organizations to learn from others, harness the impending legislation around an EU Data Space, and improve the lives of people living with diabetes in Ireland.

## Data Availability

Data sharing not applicable to this article as no datasets were generated or analysed during the current study.
